# Monitoring Gait Complexity as an Indicator for Running-Related Injury Risk in Collegiate Cross-Country Runners: A Proof-of-Concept Study

**DOI:** 10.3389/fspor.2021.630975

**Published:** 2021-05-21

**Authors:** Allison H. Gruber, James McDonnell, John J. Davis, Jacob E. Vollmar, Jaroslaw Harezlak, Max R. Paquette

**Affiliations:** ^1^Biomechanics Laboratory, Department of Kinesiology, School of Public Health – Bloomington, Indiana University, Bloomington, IN, United States; ^2^Performance Engineering Laboratory, Reebok International, Boston, MA, United States; ^3^Department of Epidemiology and Biostatistics, School of Public Health – Bloomington, Indiana University, Bloomington, IN, United States; ^4^Musculoskeletal Analysis Laboratory, College of Health Sciences, University of Memphis, Memphis, TN, United States

**Keywords:** running injury, control entropy, gait complexity, collegiate running, wearable technology in sports, accelerometry

## Abstract

Dynamical systems theory suggests that studying the complexity of biological signals could lead to a single gait metric that reliably predicts risk of running-related injury (RRI). The purposes of this pilot study were to examine center of mass (COM) acceleration complexity at baseline, prior to RRI, and the change between timepoints between collegiate runners who developed RRI during a competitive season and those who remained uninjured, and to determine if complexity at these timepoints was associated with increased odds of RRI. Twenty-two collegiate runners from the same cross-country team wore a waist-mounted triaxial accelerometer (100 Hz) during easy-intensity runs throughout the competitive season. RRIs requiring medical attention were reported via an online survey. Control entropy was used to estimate the complexity of the resultant COM acceleration recorded during each run. Associations between complexity and RRI were assessed using a frequency-matching strategy where uninjured participants were paired with injured participants using complexity from the most time-proximal run prior to RRI. Seven runners sustained an RRI. No significant differences were observed between injured and uninjured groups for baseline complexity (*p* = 0.364, *d* = 0.405), pre-injury complexity (*p* = 0.258, *d* = 0.581), or change from baseline to pre-injury (*p* = 0.101, *d* = 0.963). There were no statistically significant associations found between complexity and RRI risk. Although no significant associations were found, the median effect from the models indicated that an increase in baseline complexity, pre-injury complexity, and change in complexity from baseline each corresponded to an increased odds of sustaining an RRI [baseline: odds ratio (OR) = 1.560, 95% CI = 0.587–4.143, *p* = 0.372; pre-injury: OR = 1.926, 95% CI: 0.689–5.382, *p* = 0.211; change from baseline: OR = 1.119; 95% CI: 0.839–1.491, *p* = 0.445). Despite non-significance and wide confidence intervals that included both positive and negative associations, the point estimates for >98% of the 10,000 frequency-case–control-matched model fits indicated that matching strategy did not influence the directionality of the association estimates between complexity and RRI risk (i.e., odds ratio >1.0). This pilot study demonstrates initial feasibility that additional research may support COM acceleration complexity as a useful single-metric monitoring system for RRI risk during real-world training. Follow-up work should assess longitudinal associations between gait complexity and running-related injury in larger cohorts.

## Introduction

Human running is a complex movement that arises from the interaction and coordination of multiple codependent subsystems, including the respiratory, cardiovascular, nervous, and musculoskeletal systems. This coordination and codependence between systems makes human movement a complex dynamical systems (Glazier et al., 2003). Biological signals produced by a dynamical system are theorized to contain information about the adaptability of the system, and therefore its overall health (Golberger, 1996; Lipsitz, 2002). As the health of the system declines, these biological signals will become increasingly predictable and less complex over time as the system becomes less able to adapt to physiological stressors (Hamill et al., 1999). We refer the reader to references (Glazier et al., 2003; Gruber et al., 2011; van Emmerik et al., 2016) for an overview of the conceptual framework for dynamical systems theory.

Research into the mechanisms of running-related injuries (RRI) has led to inconsistent results, potentially because traditional analyses may be inadequate to provide insight into the complex interaction between systems that control gait. Traditional analyses focus on the association of individual system components or environmental factors to RRI, such as individual gait variables or training characteristics. However, the development of the actual tissue damage or physiological state that characterizes any specific RRI likely depends on multiple internal (anatomical, mechanical, physiological) and external (environmental, training) factors (Davids et al., 2003; Bertelsen et al., 2017). Indeed, two runners who develop an RRI may do so for different reasons. A dynamical systems approach offers an elegant solution to these individual determinants of RRI—regardless of the specific risk factor leading to RRI within an individual, all factors contribute in some way to deteriorations in the overall health of the dynamical system and therefore should alter the complexity of biological signals generated by the system (i.e., the runner). A dynamical systems approach to RRI examines the longitudinal changes in whole-body system dynamics that can indicate abrupt changes in system health or behavior of the interacting subsystems (Beek et al., 1995; van Emmerik et al., 2016), and these approaches have been used previously to identify pathological gait (Wagenaar and van Emmerik, 1994). As such, examining changes in the biological signals produced by the system—such as the acceleration of the body's center of mass—may lead to a method that can identify critical changes in running gait prior to RRI onset.

Quantifying changes in a biological signal that may indicate impending RRI requires a metric that is sensitive to complex, non-linear fluctuations. Statistical entropy is a category of non-linear dynamical systems techniques that is sensitive enough to quantify differences in physiological signal complexity (i.e., degree of regularity) between groups and physiological states. The sensitivity of statistical entropy has been observed by analyzing single variables that represent the motion of the whole body, such as center of mass and center of pressure, which could be considered to minimize the signal information from the system. Although measuring a single metric to summarize the health of a system could involve sacrificing information, statistical entropy overcomes this information loss by analyzing the behavior of a signal that represents the multiple subsystems producing or contributing to the behavior of the signal that potentially describe the system's overall health. For example, researchers have used permutation entropy to differentiate gait between people with and without cerebral palsy (Zanin et al., 2018), multiscale entropy to differentiate center of pressure between those with and without adolescent idiopathic scoliosis (Gruber et al., 2011), and sample entropy to differentiate heart rate dynamics between healthy infants and infants who were later clinically diagnosed with neonatal sepsis (Lake et al., 2002). Control entropy was developed to quantify complexity of a non-stationary time series (Bollt et al., 2009), which is appropriate for studying running in non-steady state or uncontrolled environments. For example, it has been used to determine that center of mass acceleration complexity during running is greater in trained runners than in untrained runners (Parshad et al., 2012) and decreases prior to the onset of fatigue during an exhaustive run (McGregor et al., 2009). Given that greater complexity may be a characteristic of healthy systems that possess a higher adaptive capacity (Costa et al., 2002), a decrease in complexity could potentially serve as warning of a detrimental change in a biological signal before the change physically manifests. However, much of the dynamical systems work comparing groups of different health status has been cross-sectional. Therefore, longitudinal studies are needed to test the utility of dynamical system measures for serving as an indicator for a change in health status or injury risk.

To develop RRI prevention strategies or to intervene before an RRI is sustained, a sensitive and reliable measure must be established that can assess the dynamics of a runner's gait without prior knowledge of any one potential gait or training-related risk factor. Given that center of mass motion during running is a single metric that represents the motion of the whole body, tracking the complexity of this variable alone may eliminate the need to identify a specific risk factor *a priori*. The recent availability and development of low-cost wearable technology allows center of mass acceleration to be conveniently and frequently measured outside of the lab.

While there are strong theoretical grounds to examine center of mass acceleration complexity as a potential indicator of impending RRI, research to date has not yet assessed the viability of its use in a longitudinal setting. Therefore, we aimed to test the feasibility of using center of mass acceleration complexity as a predictor of RRI in a real-world, longitudinal setting. The purposes of this proof of concept study were (a) to compare center of mass (COM) acceleration complexity at baseline, the run before a reported RRI, and the change in complexity between these timepoints between collegiate runners who developed an RRI during a competitive season and those who remained uninjured, and (b) to determine if complexity at these timepoints or its change during a competitive season was associated with an increased odds of sustaining an RRI. Collegiate runners were enrolled so that the differences in COM acceleration complexity between prospectively injured and uninjured runners could be associated with RRI risk rather than changes in running skill (Parshad et al., 2012), which should be minimal during the season in this sample. We hypothesized that (a) COM acceleration complexity would be lower at baseline in collegiate runners who developed an RRI compared with teammates who remained uninjured and (b) COM acceleration complexity measured during training runs would decrease prior to the onset of RRI and remain unchanged in controls.

## Materials and Methods

### Participants

Thirty collegiate cross-country male and female runners participated in the study (Table 1). Given evidence that running skill affects COM acceleration complexity (Parshad et al., 2012), the study design included highly skilled, well-trained collegiate runners to minimize the potential for changes in complexity that reflect skill acquisition, as opposed to changes in the health of the system (i.e., RRI). All participants gave written informed consent to participate. The protocols of this study were approved by the University of Memphis Institutional Review Board, and data sharing and analysis protocols were approved by the Indiana University Institutional Review Board. Written support to recruit and enroll participants from the testing site's University cross-country/track and field team was provided by the head coach.

**Table 1:** Subject demographics.

	**Uninjured**	**Injured**
Age, years	19.79 ± 1.35	18.71 ± 0.95
Height, cm	177.07 ± 9.06	173.81 ± 11.06
Mass, kg	63.37 ± 7.73	59.49 ± 9.36
Sex	Male 11; female 4	Male 3; female 4
Follow-up, weeks	10.0 ± 1.9	3.3 ± 3.8
Recorded runs, #	251	32

*Values are group mean ± 1 standard deviation. Follow-up period was the number of weeks between baseline and the run recorded before a running related injury was sustained in the injured group (i.e., ‘pre-injury'). In the uninjured group, the follow-up period was the number of weeks between the baseline run and the last recorded. The number of recorded runs was the number of runs completed with the accelerometer across participants within each group, during the respective follow-up periods*.

### Protocol

**Figure 1:**
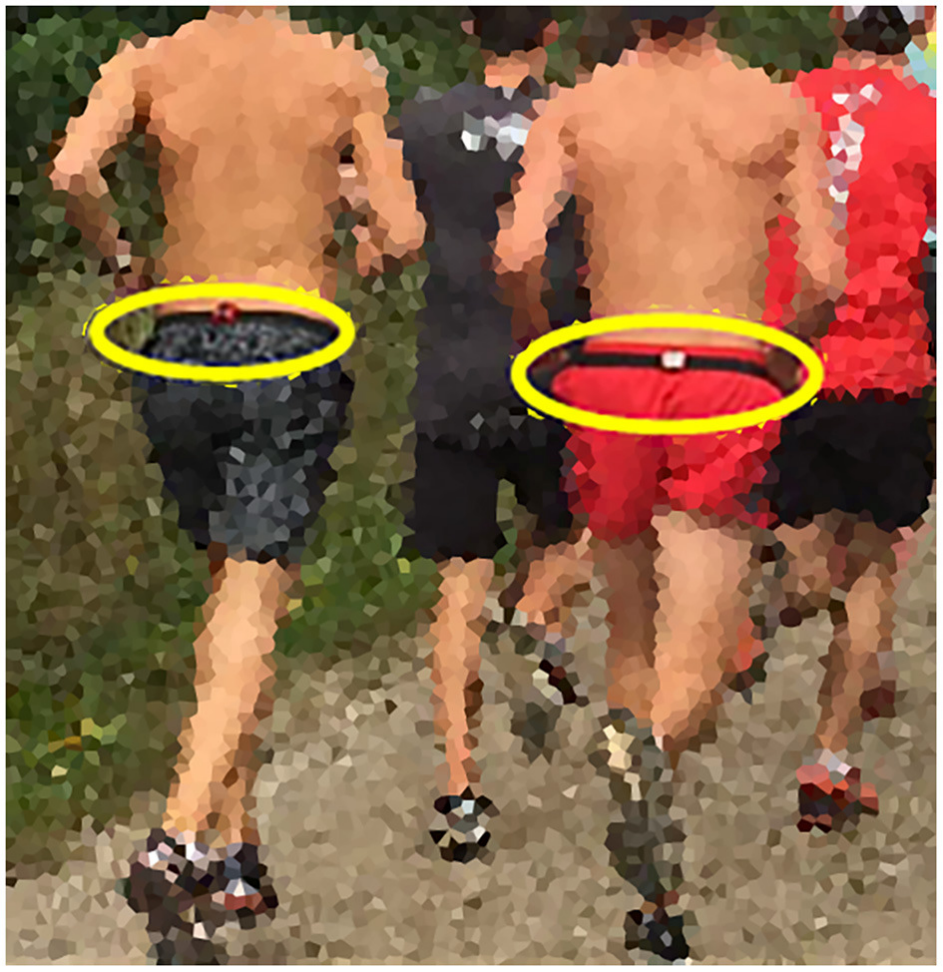
The triaxial accelerometer (GT3+, ActiGraph Corp, Pensacola, FL, USA; 100 Hz) was positioned securely around the waist, centered over the sacrum. Participants wore the device for all “easy” continuous runs each week of the competitive season. The group baseline run is pictured in the figure.

All participants were given a triaxial accelerometer (GTX3+, ActiGraph Corp, Pensacola, FL, USA) at the beginning of their competitive collegiate cross-country season (i.e., early September). The accelerometers were initialized to collect acceleration data continuously at 100 Hz (output unit = g; range ± 6 g). Participants were instructed to wear the accelerometer over the posterior aspect of the proximal sacrum, secured using an adjustable elastic belt (Figure 1). The belt was tightened so the accelerometer would not bounce or rotate from its position over the pelvis. On the first day of follow-up, all participants completed a baseline run, which was an easy run on a known cross-country loop that included multiple surface types (pavement, grass, packed dirt, and wood chips). As prescribed by the coaches, the participants ran at an easy intensity, but the duration of the baseline run varied among participants because each trained at similar, but not identical, weekly running distances.

After the baseline run, participants were instructed to wear the accelerometer during all continuous easy training runs and weekend long runs throughout the fall cross-country season. An example of a typical week of training is presented in Table 2. Coaches instructed the runners to complete easy-intensity runs, including one long run, on ~3 to 4 days per week during the season. These runs were performed on a familiar course at the athlete's preferred light/easy and relatively constant aerobic pace, ranging between 30 and 60 min. A longer easy run was included as one of the three to four easy-intensity runs prescribed per week. This long run was performed at a similar effort but for a longer duration (typically 65–110 min). The accelerometer was not worn during interval running sessions (i.e., workouts) given the large changes in running speeds performed during these sessions and was not worn during races to minimize participant burden in this proof-of-concept study.

**Table 2:** Example of a typical training week prescribed by the team head coach.

	**Monday**	**Tuesday**	**Wednesday**	**Thursday**	**Friday**	**Saturday**	**Sunday**
a.m.		Easy run [or cross training]		Easy run [or cross training]	Rest day	Race or easy run or cross train or rest	Long run
p.m.	Workout + plyometrics	Cross training + strength [or easy run]	Workout + plyometrics	Cross training + strength [or easy run]			

*Participants were free to select the mode of cross-training, which typically included swimming, cycling, elliptical, or similar. Participants were instructed to complete only one “easy run” on Tuesdays and Thursdays. That is, an easy run would be performed either in the morning or in the afternoon, but not both. The accelerometer was worn during all easy and long runs throughout the fall cross-country season (approximately three to four runs per week for 14 weeks). Most participants completed a race on Saturdays and may have taken 1 day per week off from training; therefore, accelerometer wear compliance was calculated using three runs per week as the prescribed training*.

All participants followed the same training program prescribed by the same coach with respect to the following: timing and days of training sessions; the type of run for each training session (e.g., interval, tempo, easy run, long run, etc.); the intensity of a given training session (i.e., workouts were high relative intensity, continuous runs were low relative intensity); training routes and/or terrains; in-season racing schedule; and similar recovery strategies (e.g., massage, foam rolling, ice baths, etc.). Thus, the absolute time and or distance for a given run or week of running was different among runners, but a similar relative training intensity was prescribed across all participants.

For the duration of the cross-country season, each participant was instructed to report musculoskeletal pain or discomfort to the athletic training staff. Participants reported the date of the pain occurrence or injury, location, and severity of the pain or discomfort on a 0–10 scale using a mobile survey app developed for the study. Participants were allocated to the injured group if (a) they sought medical treatment from the athletic training staff (Davis et al., 2016) and reported experiencing a pain level ≥4 or (b) the athletic trainer gave a specific diagnosis. The most recently observed run recorded by the accelerometer (at least 1 day prior to the onset of injury) was designated as the “pre-injury” timepoint in the analysis. If a participant reported an RRI before a second run was recorded or on the same day as the second recorded run, then only the baseline run would be included in the analysis. That is, for these participants, the same value would be used for baseline and pre-injury complexity because the most recent estimate of their complexity would be the baseline run.

Eight out of the initial 30 participants were removed from the analysis after the study was completed: one participant lost the accelerometer, and seven participants had complaints of pain or fatigue that could not be related to a specific cause or was not reported in the survey. The final sample used for analysis (*n* = 22) included 15 uninjured controls and seven participants who met the above criteria for allocation into the injured group.

### Data Analyses

The raw, three-dimensional COM acceleration signal recorded over the duration of the study period was downloaded from each accelerometer for analysis. The resultant (vector magnitude) of the acceleration signal was calculated using the Euclidean norm and was used for all subsequent analyses. For each recorded run, a custom activity recognition algorithm developed in MATLAB version R2020a (The MathWorks Inc, Natick, MA, USA) was used to identify and extract periods of non-running data from the running time-series data (Davis IV et al., 2019). For example, if a participant briefly stopped (e.g., tie a shoe) or walked during his/her run, this non-running time was isolated and removed from the time series. Data identified as “running” with this algorithm had to last 30 s or more for it to remain in the time series. All periods identified as running were appended together and treated as a single continuous run. The total duration of the analyzed time series from each individual running session was between 17,900 and 665,500 data points (i.e., 2.98–110.92 min).

### Control Entropy Calculation

**Figure 2:**
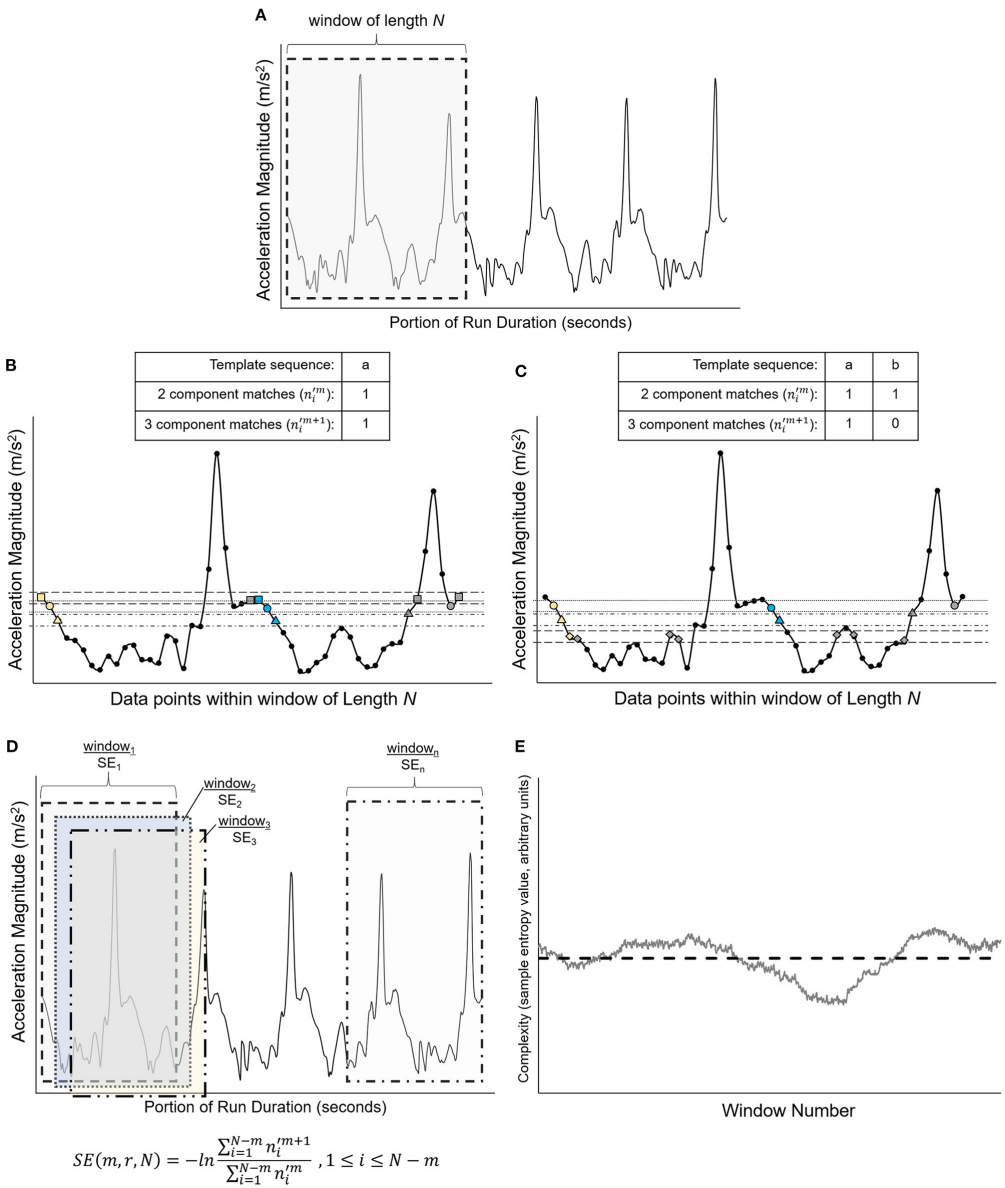
Graphical representation of the vector matching procedure and sample entropy calculation [adapted from Costa et al. (2005)]. **(A)** Shows a portion of a simulated time series extracted from a simulated time series, with data points *x*_1_, …, *x*_N_. The first overlapping window of length *N* is represented by the gray-shaded region and is isolated in **(B)**. **(B)** Shows the first template sequence (□, °, Δ), which consists of data points *x*_1_, *x*_2_, and *x*_3_ and is represented by the yellow symbols. The horizontal dashed lines represent the similarity threshold *r* around each data point of the template sequence. Two data points will match if the absolute difference between them is ≤ *r*. Blue-filled symbols are data points that fall within the similarity threshold *r* and match the sequence of data points for *m* and *m*+1 vector lengths of the template (i.e., for m = 2, vector length *m* is represented by □, ° and vector length *m*+1 is represented by □, °, Δ). The gray-filled symbols are data points that are within the similarity threshold *r* but do not match the vector sequence (□, ° or □, °, Δ). The numbers of *m* and *m*+1 matches are counted [one match for each vector length for the sequence shown in **(B)**], then the template sequence is shifted by one data point to data points *x*_2_, *x*_3_, and *x*_4_, as shown in **(C)**. The template sequence in **(C)** (°, Δ, ◇) has one match for vector length *m* (°, Δ) and zero matches for vector length *m*+1 (°, Δ, ◇). As shown in **(D)**, the process is repeated within the window length *N* for each successive template sequence until the first data point of the template sequence is *x*_N−m_. The sample entropy (SE) for each window length *N* is calculated as the negative natural logarithm of the ratio of three component matches to two component matches. For the equation in **(D)**, the numerator is the sum of the three component matches ($n_{i}^{\prime m+1}$) for each template sequence within window, and the denominator is the sum of the two-component matches ($n_{i}^{\prime m}$) for each template sequence within window. **(E)** Shows the result of this procedure, which is a time series of sample entropy values for each window of length *N* across the entire time series, which was a single running bout for the present study. The mean complexity for each run was quantified by calculating the average of these sample entropy values across the duration of the run, represented graphically by the horizontal dashed line (Costa et al., 2002, 2003, 2005; Bollt et al., 2009; Costa and Goldberger, 2015).

A custom MATLAB program incorporating the Physionet Toolbox (Goldberger et al., 2000) was used to perform the control entropy analysis on the unfiltered, resultant COM acceleration signal of the extracted runs. Detailed methods of the control entropy technique are described elsewhere (e.g., Bollt et al., 2009; McGregor et al., 2009; Busa and van Emmerik, 2016). The method is presented graphically in Figure 2. In brief, the control entropy procedure involves calculating the sample entropy of the center of mass acceleration signal within discrete overlapping (i.e., sliding) windows of length *N*, where *N* = 750 data points (7.5 s). Sample entropy assesses the probability that two similar vectors of *m* data points will remain similar with the addition of a consecutive data point, *m* + 1 (Costa and Goldberger, 2015). Window length *N* = 750 was selected because it may be an optimal window size to reveal time-dependent fluctuations in the biological signal without loss of signal variability from excessive smoothing (Costa et al., 2003; Bollt et al., 2009; Zhou et al., 2017). The SampleEntropy(*m,r, N*) function from the Physionet toolbox was used to calculate this probability by determining the number of similar vectors of length *m* and of length *m* + 1 that repeat within the windowed time series of length *N*. The vectors are recognized as similar and repeating if the corresponding sequence data points in each vector fall within tolerance *r*, where *r* is a percentage of the standard deviation of the time series (*r* = 0.15 for this study). Next, the sum of repeating vectors of length *m* + 1 is divided by the sum of repeating vectors of length *m*. The negative natural log of this ratio is the sample entropy. The calculation is performed as follows:

$$S_{E}\left(m,r,N\right)=-ln\frac{\sum_{i=1}^{N-m}n_{i}^{\prime m+1}\;}{\sum_{i=1}^{N-m}n_{i}^{\prime m}\;},\;1\leq i\leq N-m$$

where $n_{i}^{\prime m}$ is the number of vector matches for vectors with length *m* and $n_{i}^{\prime m+1}$ is the number of vector matches for vectors with length *m*+1 (Costa et al., 2005). The procedure results in a time series of sample entropy values for each overlapping window of 750 data points. Lower sample entropy values occur when the ratio between *m* + 1 vector matches and *m* vector matches is closer to 1, which represents a time series that is more repeatable. Greater sample entropy values occur when the ratio of *m* + 1 vector matches and *m* vector matches is closer to 0, which represents a time series that is more random (Lake et al., 2002). In this study, parameters of *m* = 2, *r* = 0.15, and *N* = 750 were used (Costa et al., 2005; Zhou et al., 2017). The average of these sample entropy values is the complexity of the signal for a given single running bout.

### Statistical Analysis

Accelerometer wear compliance was calculated for each participant as the number of runs recorded relative to the estimated minimum number of scheduled easy-intensity runs between the baseline run and the last recorded run (i.e., three easy-intensity runs per week, equating to an estimated minimum total of 522 scheduled runs across all analyzed participants and weeks between baseline and the last recorded or pre-injury run). The last recorded run for the injured group was the last run recorded prior to the reported RRI. The number of weeks between the baseline run and the last recorded run was rounded to half-week increments, and the number of estimated scheduled runs was rounded to the nearest whole number prior to compliance calculation. Compliance was calculated for each participant, then the mean and standard deviation was calculated for each group.

For each injured participant, two control entropy values were considered for analysis: the mean control entropy of the participant's first recorded run (“baseline complexity”) and the mean control entropy of the run recorded by the accelerometer at least 1 day prior to the date of reported RRI (“pre-injury complexity”). Two participants reported an RRI on the same day as their second run recorded, and so the baseline run was used for baseline and pre-injury complexity. A frequency-matching technique was used to randomly select runs from two uninjured participants that corresponded with the pre-injury run of each injured participant. That is, two uninjured runners were randomly selected to provide a baseline complexity and a “date-matched” pre-injury complexity value for comparison to each injured runner's pre-injury complexity value. The runs selected from the uninjured participants were required to be recorded by the accelerometer within ±7 days of the pre-injury date of the injured participant. If no runs were recorded during this window, the uninjured comparison participant was returned to the pool of possible uninjured matches, and another match was drawn. This process was repeated for all injured participants. With this method, the pre-injury complexity values for each of the seven injured participants were randomly matched to two corresponding complexity values from uninjured participants, taken at similar times during the cross-country season. This frequency-matching strategy randomly selected 14 of the 15 uninjured runners to pair with pre-injury values in the injured participants; the 15th uninjured runner provided an additional pre-injury complexity value that was selected in a ±7-day window on a randomly selected date between the first and last dates of RRI observed in the injured group.

To ensure that the results from this frequency-matching strategy were not contingent on one particular random sampling of uninjured participants or dates, we replicated this frequency-matching strategy 10,000 times, fitting statistical models to each of these data replicates. In 22% of cases, no matching runs within the randomly drawn ±7-day window were found for the final 15th uninjured participant. We conducted a sensitivity analysis in which the participant with the failed match was excluded in that replicate and an analysis in which any replicates with a failed match were completely ignored. Effect estimates changed by <5% in all models when comparing these two possible strategies, indicating that this choice had very little influence on our results. As such, in the cases of failed matches within replicates, we excluded the uninjured participant whose match had failed for that replicate only. We summarize the results of these replicates for all models by reporting the median of the effect estimate for each model (e.g., the odds ratio or the linear regression slope) as well as the *p*-value and 95% confidence intervals calculated from the model with the median effect estimate, across all 10,000 models fit for each research question. All statistical analyses were conducted in MATLAB version R2020a.

Group differences in complexity at baseline and pre-injury were estimated using a linear mixed-effect model fit to each of the 10,000 frequency-case–control-matching replicates, with a random intercept term for each participant to account for the repeated-measure nature of the observations (Fitzmaurice et al., 2011). The median effect estimates across these replicates were used to summarize group differences at baseline and at pre-injury. To compare simple group differences in complexity at each timepoint, Cohen's *d* effect size was also calculated for baseline complexity, pre-injury complexity, and change from baseline complexity, with thresholds of 0.2, 0.5, and 0.8 for small, medium, and large sizes, respectively (Cohen, 1988). The effect size results for each of the matching iterations are summarized in the Supplemental Material.

We used logistic regression to examine the association between complexity and RRI risk at baseline and immediately prior to injury for which the odds ratios were reported. Pre-injury complexity was assessed both in absolute terms and when expressed relative to change in baseline. We used linear regression to examine differences in pre-injury complexity between the injured and uninjured groups, after adjusting for baseline complexity.

## Results

A total of 283 runs were recorded by the 22 participants included in the analysis out of a minimum estimated 522 scheduled runs. The mean ±1 standard deviation compliance of the injured group was 67.9 ± 30.1% and was 55.9 ± 20.8% for the uninjured control group (Table 1). The pre-injury run was recorded an average of 4.6 ± 3.1 days prior to the onset of injury among the injured runners. Two participants reported an RRI on the same day as their second run was recorded, which was 2 and 5 days after the baseline run. The location of the reported injuries included anterior leg, ankle, low-back/hip, and thigh, and two participants reported foot injuries. The participants who received a specific diagnosis (*n* = 5) self-reported the following: medial tibial stress syndrome, foot muscle strain, hip adductor strain, plantar fasciitis, and ankle instability and a pain level of 7.4 ± 0.9. The two additional participants who sought treatment by the athletic trainers reported experiencing a pain level of 4 and 7 out of 10 but did not self-report a specific diagnosis.

**Figure 3:**
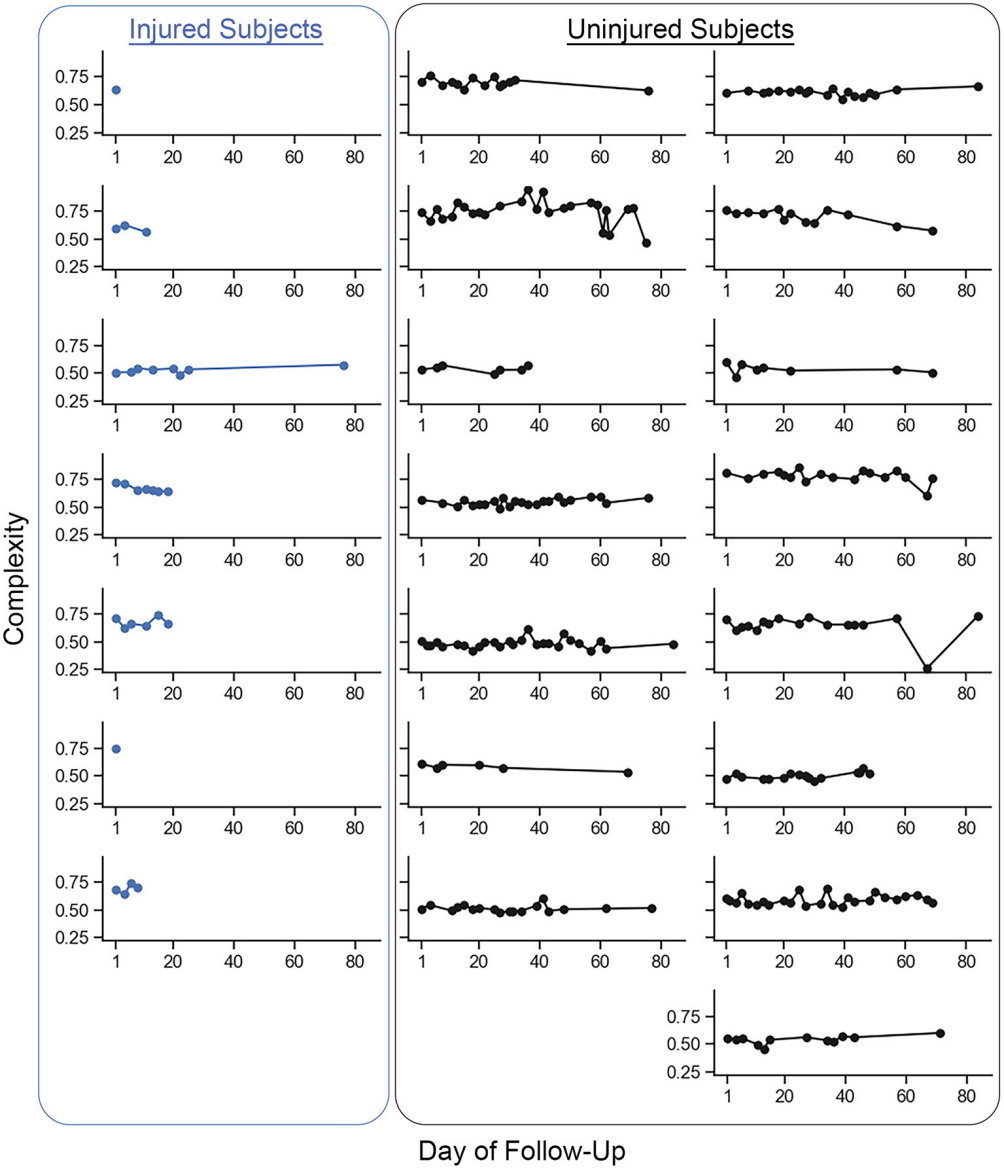
Individual mean complexity values for each recorded run across participants from the baseline run to the last recorded run. Each dot represents the mean complexity value of a single recorded run from an individual participant. The injured group participants are represented in the column of plots on the left. All other plots are the uninjured group participants. The last recorded run for the injured group was the last run recorded prior to the reported running-related injury (i.e., ‘pre-injury'). Two participants in the injured group reported a running-related injury prior to recording a second run and so only one run is presented. Changes in complexity within participants were small on a per-unit basis (mean ± 1 standard deviation change (i.e., pre-injury minus baseline) in run-to-run complexity within a given participant was −0.0031 ± 0.0085 units. The between-participant standard deviation (0.0871 units) in run-to-run complexity magnitude was more than 60% larger than the within-participant standard deviation (0.0529 units).

**Figure 4:**
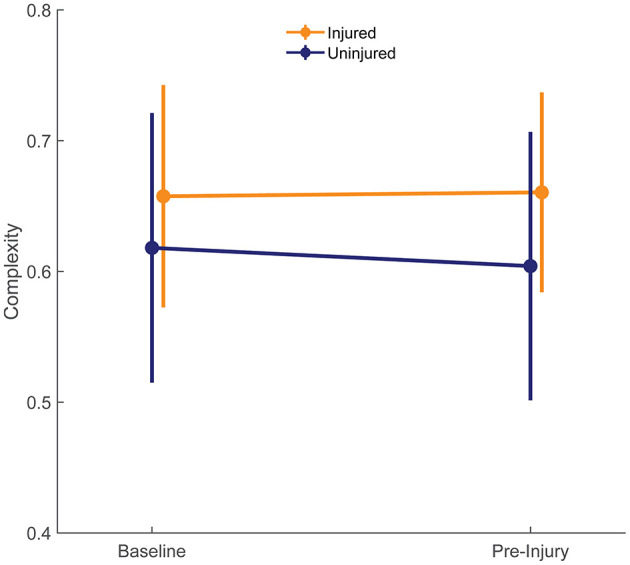
Complexity at baseline of both groups is compared with the last run prior to injury (pre-injury) in injured group. The pre-injury values for the uninjured runners were the median of the corresponding date-matched run of the uninjured control group obtained from the frequency matching procedure (i.e., the medians of each estimate across the 10,000 random pairing replicates). Error bars show ± one standard deviation of the group mean.

Complexity values for each run are presented in Figure 3. Figure 4 compares mean baseline and pre-injury complexity of the injured group with date-matched runs in the uninjured group. The provided estimates for the uninjured group are the median estimates across replicates for the pre-injury date matching strategy, which mitigates the effects of any one particular chance pairing of injured to uninjured control subjects.

At baseline, the uninjured group had an average complexity value of 0.618 ± 0.103 units compared with 0.658 ± 0.085 units in the injured group. These differences were small in size (*d* = 0.405) and not statistically significant (95% CI: −0.045 to 0.124, *p* = 0.364; Figure 4). At pre-injury, the uninjured group's complexity had decreased by 0.014 units, while the injured group's complexity had increased by 0.003 units. The effect size for the group difference in pre-injury complexity was moderate (*d* = 0.581), and the effect size for the group differences in change from baseline complexity was large (*d* = 0.963). However, change in entropy from baseline was not significantly different between groups (injury-by-time interaction effect 0.017, 95% CI: −0.012 to 0.047, *p* = 0.258).

**Figure 5:**
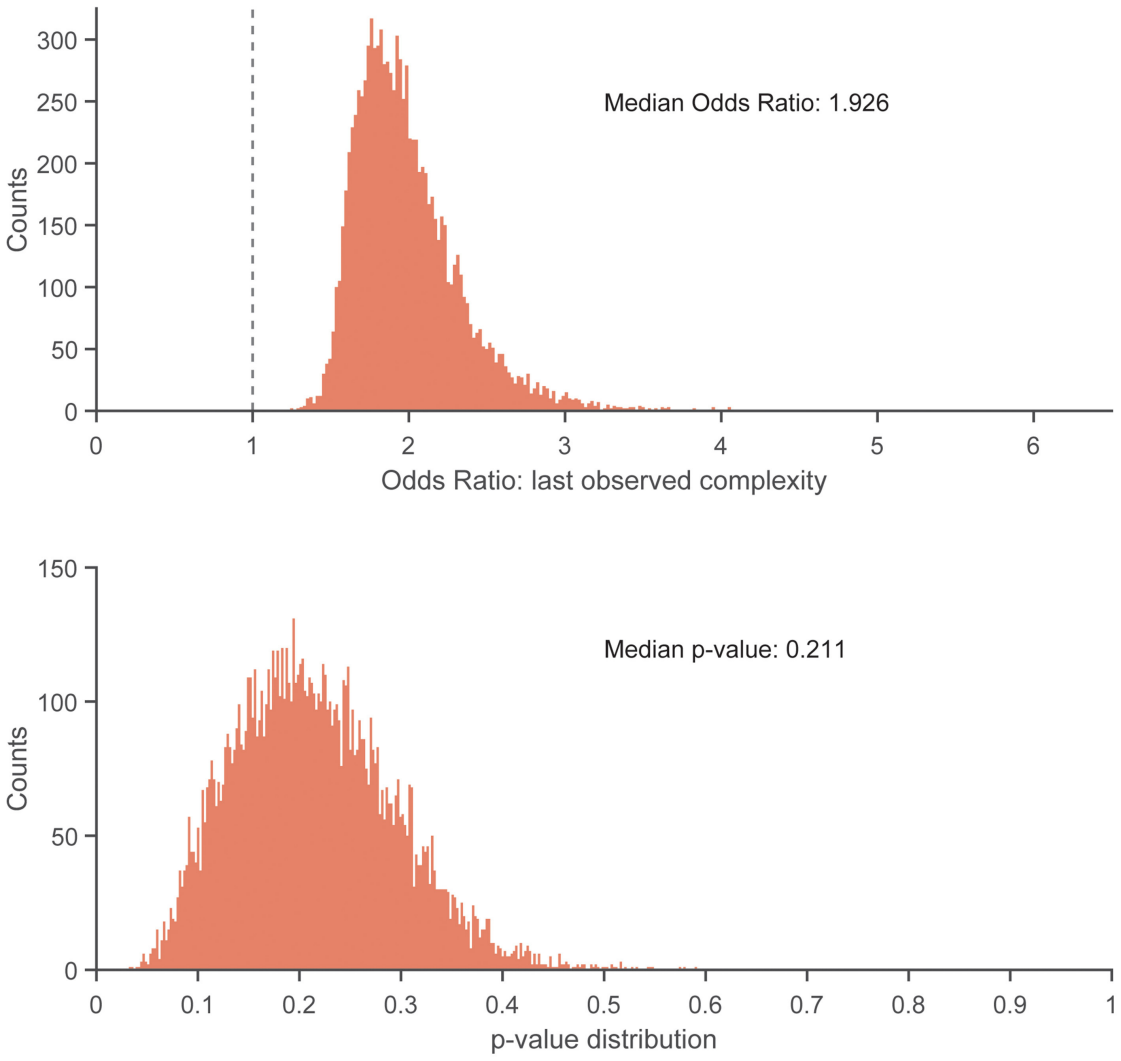
Histogram showing distribution of odds ratios **(Top)** and *p*-values **(Bottom)** for the increase in injury odds per 0.1-unit increase in pre-injury complexity, across 10,000 replicates of the frequency-matching procedure. “Pre-injury” complexity was the last observed complexity value prior to reported injury in the injured group and the date-matched run of the control group resulting from the frequency-matching procedure. The vertical dashed line in the top panel indicates the null hypothesis of no association (odds ratio = 1.0). 100% of the replicates resulted in an odds ratio > 1.0.

**Figure 6:**
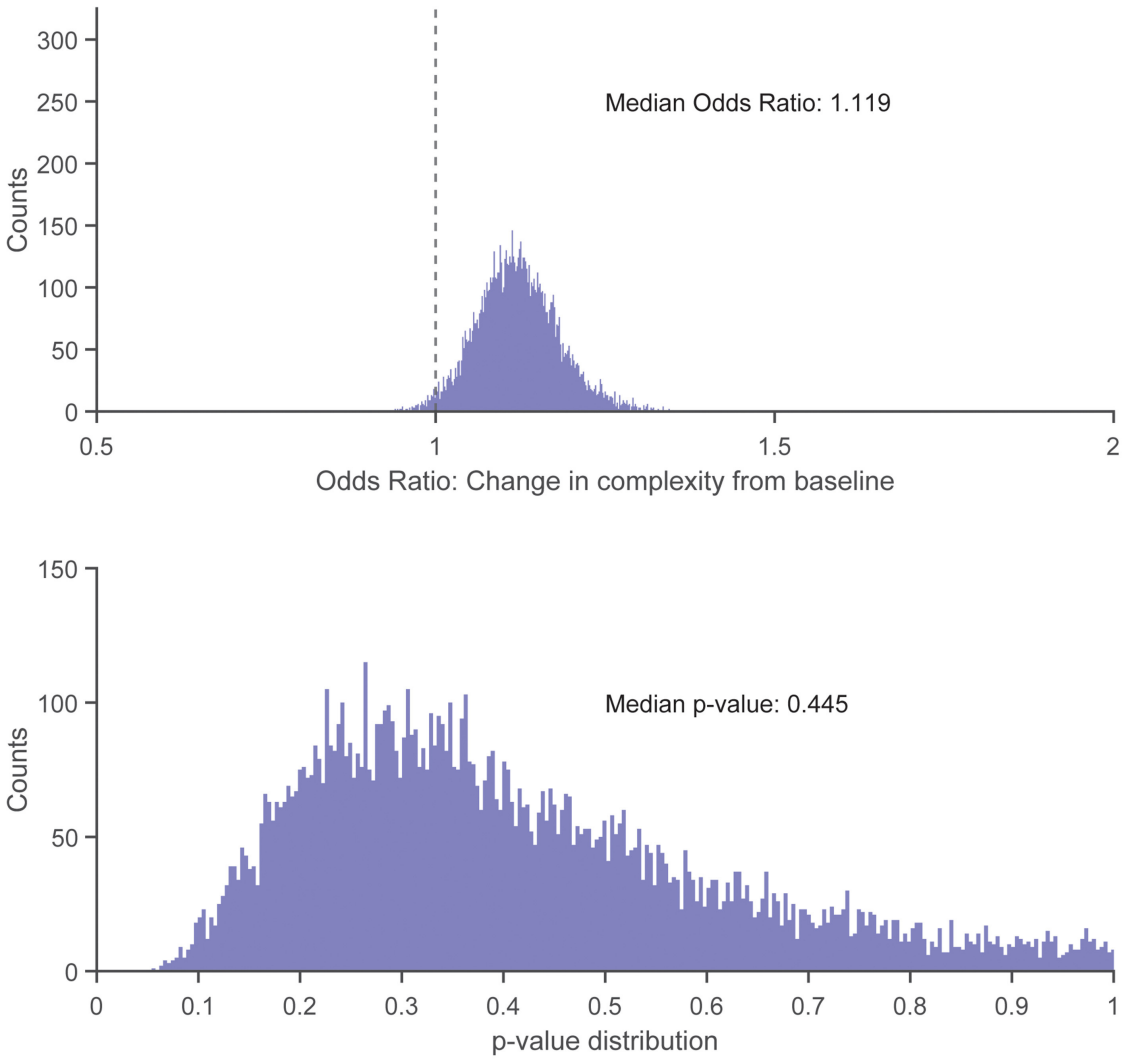
Histogram showing distribution of odds ratios **(Top)** and *p*-values **(Bottom)** for the increase in injury odds per 0.01-unit increase in change from baseline complexity (calculated as pre-injury complexity minus baseline complexity), across 10,000 replicates of the frequency-matching procedure. “Pre-injury” complexity was the last observed complexity value prior to reported injury in the injured group and the date-matched run of the control group resulting from the frequency-matching procedure. The vertical dashed line in the top panel indicates the null hypothesis of no association (odds ratio = 1.0); 98.27% of the model replicates resulted in an odds ratio > 1.0.

**Figure 7:**
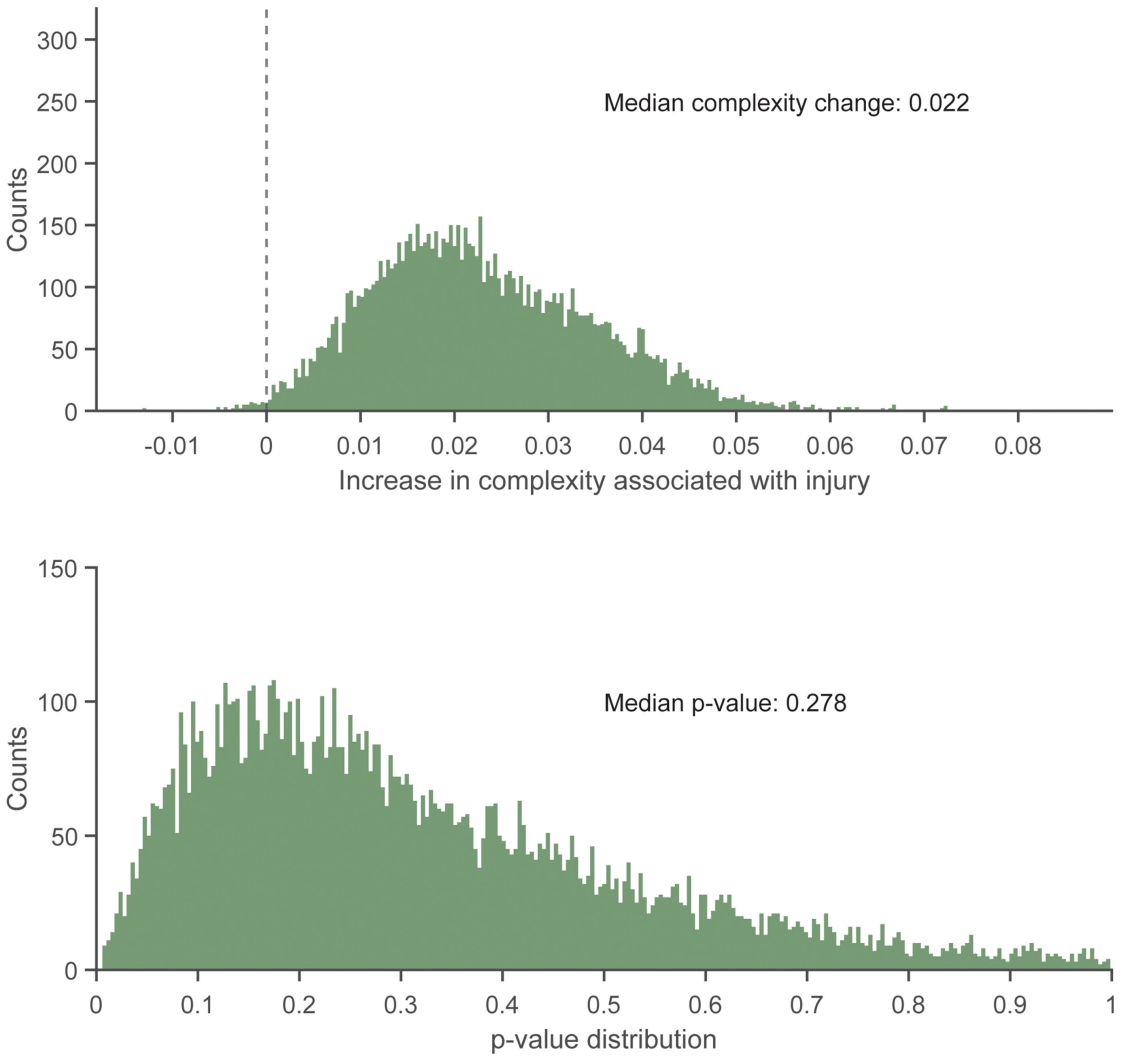
Histogram showing the distribution of regression slopes **(Top)** and *p*-values **(Bottom)** for the increase in complexity in the injured group vs. the uninjured group at pre-injury, after adjusting for baseline complexity, across 10,000 replicates of the frequency-matching procedure. “Pre-injury” complexity was the last observed complexity value prior to reported injury in the injured group and the date-matched run of the control group resulting from the frequency matching procedure. The vertical dashed line in the top panel indicates the null hypothesis of no association (injured group increase in complexity vs. uninjured group = 0.0). That is, an effect >0.0 is positive, indicating greater complexity in the injured group. 99.37% of iterations had effect estimates for the injured group of >0.0.

There were no statistically significant associations found between control entropy and the risk of RRI. Although no significant differences were found, the point estimates for >98% of the 10,000 frequency-matched iterations indicated that matching strategy did not influence the directionality of the association estimates between complexity and RRI risk (i.e., odds ratio >1.0) (Figures 5–7). The median effect estimate across models of baseline complexity indicated that each 0.1-unit increase in baseline complexity corresponded to a 1.560-fold increase in odds of sustaining an RRI (95% CI: 0.587 to 4.143, *p* = 0.372). The median effect estimate across models of pre-injury complexity indicated that each 0.1-unit increase in pre-injury complexity corresponded to a 1.926-fold increase in odds of sustaining an RRI (95% confidence interval 0.689 to 5.382, *p* = 0.211; Figure 5). When considering change from baseline complexity, the median effect estimate across models indicated that each 0.01-unit increase at pre-injury corresponded to a 1.119-fold increase in odds of sustaining an RRI (0.839 to 1.491, *p* = 0.445; Figure 6). In the injured group, pre-injury complexity was 0.022 units greater than in the controls, after adjusting for baseline complexity (95% CI: −0.016 to 0.059, *p* = 0.278; Figure 7).

## Discussion

The purposes of this proof-of-concept study were (a) to compare COM acceleration complexity at baseline, the run before a reported RRI (“pre-injury”), and the change in complexity from baseline to pre-injury between collegiate runners who developed an RRI during a competitive season and those who remained uninjured and (b) to determine if complexity at these timepoints or its change during a competitive season was associated with increased odds of sustaining an RRI. Contrary to our hypothesis, the difference between baseline COM acceleration complexity in the injured group and the complexity prior to the onset of RRI was not significant. Also, the injured and control groups had statistically similar baseline and pre-injury complexity, with the injured group having slightly larger complexity magnitudes than controls (Figure 4). Although there were no statistically significant associations found between complexity and risk of RRI, the near-consistent direction of the matching replicates and the moderate to large effect size results provides initial feasibility and support of our approach: investigating COM acceleration complexity as a potentially useful tool for monitoring RRI risk during running training may be a promising avenue for future research. This support is consistent with the purpose of pilot studies (Leon et al., 2011). If a true association between COM acceleration complexity and RRI exists, then significant associations will be revealed with future research.

The near-consistent direction of the association estimates demonstrate that the findings were irrespective of the participant matching. This consistency was found across different measures of a potential RRI–complexity relationship (baseline complexity, pre-injury complexity, and change from baseline complexity). The results of the matching replicates should be considered collectively as one result, rather than focus on the statistical outcome of each individual model result. For example, consider an alternate scenario for the model replicate results in which the median centered at an odds ratio of 1.0 (i.e., no risk of RRI with respect to complexity). That is, the distribution of matching replicates centered at an odds of 1.0 would indicate no correspondence between complexity and injury risk. This scenario would occur if the potential association between complexity and RRI risk was random or depended on the matching strategy between injured and uninjured participants. However, we found that at least 98% of the normally distributed model outcomes were above an odds ratio of 1.0 (Figures 5, 6) and indicated an increased, albeit non-significant, odds of RRI with increased complexity. Additionally, the moderate to large effect size results (Supplemental Material) indicate that the difference in change from baseline complexity may have clinical relevance, despite the non-significant *p*-value from the model. Therefore, the non-significant *p*-values may be due to either a large variance in the data or a small sample size. These results indicate initial feasibility of the premise for measuring COM acceleration complexity as a tool for monitoring RRI risk although we could not establish evidence for or against an association of complexity with RRI because no significant associations were found.

Future research in the longitudinal monitoring of COM complexity may lead to a warning system for impending RRI. Given that the increased odds of sustaining an RRI with increasing complexity were non-significant with our sample size, we make this projection cautiously. The uncertainty exhibited by large confidence intervals prevents establishing a clear direction and magnitude of change in complexity that may be associated with RRI development. Additional research with a larger sample size, more frequent measurements, and a longer monitoring period may reveal statistically significant findings regarding COM acceleration complexity and RRI risk in future research. Such an approach would address our uncertainty about whether any potential relationship between complexity and RRI is a function of baseline complexity (and thus relating to between-subject differences) or a function of acute changes in complexity (and thus related to within-subject changes).

The present study is one of the first to investigate longitudinal changes in the complexity of a gait-related biological signal in runners using a dynamical systems metric and attempt to associate it with a change in impending RRI. Current dynamical systems theories suggest that reduced complexity may represent a less adaptive and unhealthy biological system that is susceptible to injury (Hamill et al., 1999). Although the difference was non-significant, the direction for which complexity changed between baseline and pre-injury observed in the injured group was contrary to this theory. If future studies show a significant increase in complexity with RRI risk, rather than a decrease in complexity, then support for optimal ranges of complexity may be indicated, which has been proposed by others (Hamill et al., 1999; Stergiou and Decker, 2011). That is, both too much complexity and too little complexity may be harmful. We expect that an increase in complexity indicating injurious complexity above the theorized optimal threshold may only occur if the participants are highly trained, as in the present study, given that greater complexity is associated with greater running skill (Parshad et al., 2012). Similarly, the small decreases in complexity observed within the uninjured controls in future studies may represent changes that remained within the optimal threshold, given that these participants did not sustain an RRI. The contrast between the non-significant observations from our longitudinal pilot study with the results from cross-sectional dynamical systems studies is worthy of examining with larger samples and additional runner populations.

This study used control entropy as an estimate for gait complexity. Several other entropy techniques and other non-linear metrics have been used to monitor changes in human dynamical systems. For example, various entropy techniques have been used to detect altered movement patterns (Deffeyes et al., 2009a), differentiate between healthy and unhealthy heart rate dynamics (Lake et al., 2002; Costa et al., 2005), differentiate between rested and fatigued mental states (Mu et al., 2017), and detect improvements in skill (Kawazoe et al., 2009). In the context of running, sample entropy has been used to compare the effects of fatigue on trunk and tibial acceleration in runners with medial tibial stress syndrome and healthy controls (Schütte et al., 2018). Additionally, control entropy has been previously demonstrated to detect differences in the complexity of runners' COM acceleration signal when there are differences in skill or fatigue between runners (McGregor et al., 2009; Parshad et al., 2012). Alternative methods, such as detrended fluctuation analysis, have been used to associate changes in the stride interval of running with acute and chronic fatigue (Meardon et al., 2011; Fuller et al., 2017), to differentiate between trained and untrained runners (Nakayama et al., 2010), and to differentiate between retrospectively injured and uninjured runners (Meardon et al., 2011). Although detrended fluctuation analysis has many benefits, it may have a limited ability to detect changes in a system's peripheral sensory feedback (Gates and Dingwell, 2007), which may be important for prospective RRI development. Each method has benefits and drawbacks and may have differing sensitivity to various aspects of gait such as speed, incline, or terrain. Additionally, differences in complexity between groups may be task dependent (Vaillancourt et al., 2004). Selection of a non-linear method should be made carefully to ensure it is appropriate for the specific signal characteristics and the specific application. In this study, control entropy was selected to detect changes in RRI risk because of the previously demonstrated sensitivity of statistical entropy techniques for detecting differences in physiological states or health status for various physiological signals (e.g., Lake et al., 2002; Costa et al., 2005; Deffeyes et al., 2009a; Kawazoe et al., 2009; Gruber et al., 2011; Mu et al., 2017; Zanin et al., 2018), for its success in quantifying complexity of non-stationary acceleration signals of runners (Bollt et al., 2009; McGregor et al., 2009; Parshad et al., 2012), and for its noted robustness in comparing pathological and healthy signals (Cirugeda-Roldán et al., 2012).

### Limitations and Recommendations for Future Research

The control entropy calculation was chosen due to the non-stationary conditions that may arise within a free-living run. However, a limitation of the control entropy method is that it assesses complexity at a single timescale (Bollt et al., 2009; Busa and van Emmerik, 2016). Quantifying complexity over multiple timescales may be necessary to distinguish between random noise and highly complex signals (Costa et al., 2002) as well as assess fluctuations in the physiological processes that operate at varying timescales (Turvey et al., 1982; Bernstein, 1996; Busa and van Emmerik, 2016). Although multiscale entropy is an available method to quantify complexity over multiple timescales, it may be insufficient to assess non-stationary signals (Bollt et al., 2009), such as COM acceleration measured during free-living running. Incorporating multiple window lengths *N* has been suggested to address this limitation (Bollt et al., 2009) and should be assessed in future laboratory-based studies before implementing in real-world settings.

Acceleration of the COM was the variable selected for this study because it is a single variable that represents the motion of the whole body. However, some systems may adapt to maintain a constant COM acceleration pattern as a result of impending pain or injury. This adaptation may be difficult to detect, which could explain the non-significant results of the present study. However, complexity and other non-linear dynamic methods are sensitive measures for detecting changes in the behavior of the interacting subsystems that control these adaptations, even when the movement pattern is similar between clinical groups (Deffeyes et al., 2009b; Gruber et al., 2011; Bisi and Stagni, 2016). Therefore, we expect that any adaptations to maintain a specific movement pattern in the presence of pain or injury would manifest as an increase in regularity and potentially be detectible with a complexity or another non-linear dynamic analysis.

The ability to detect differences between groups may have been affected by the run we selected to compare to baseline. Complexity during the run prior to RRI was assessed to identify critical changes in running gait before the signs and symptoms of an RRI became apparent. Comparing the run from the same day as the reported RRI may have resulted in larger differences in complexity between groups, increasing the likelihood of detecting statistical significance.

The present study was more ecological than a lab-based study because it was conducted in a free-living environment. Using the control entropy calculation, rather than other entropy calculations, helps to mitigate some factors that may affect complexity magnitude in this highly ecological setting that is representative of free-living runners. However, future studies could benefit from studying more controlled environments (e.g., consistent terrain, running pace, duration, and running route) to better understand daily, run-to-run, or within-run variations in COM acceleration complexity. Differences or changes in COM acceleration complexity have previously been observed between treadmill and overground running (Lindsay et al., 2014), at the onset of fatigue, and with changes in gait speed (McGregor et al., 2009). Therefore, the influence of these typical environmental attributes must be established to improve the classification accuracy of the changes in complexity associated with impending RRI.

Mean control entropy was calculated and compared across easy runs and not during intense training sessions or races. Although detecting changes in complexity across a competitive season was the goal of the present study, more intense runs, which have been shown to cause acute changes in complexity at the onset of fatigue (McGregor et al., 2009), may be necessary to better detect the changes in COM acceleration complexity that indicate a change in RRI risk. Additionally, the pace of the baseline and pre-injury runs could have differed between runs. It is possible that the pace of the recorded runs increased slightly as the season progressed, which may have influenced COM acceleration complexity apart from changes in complexity due to impending RRI. Differences in complexity were detected previously between several walking and running speeds that differed by 2 km/h (McGregor et al., 2009). However, the coaches made it a priority that runners maintain an “easy” pace (i.e., based on perceived effort) for the recorded running sessions throughout the season. Therefore, we expect changes in pace during the season to be minimal.

Seeking medical treatment was used in current study and others (Davis et al., 2016) to allocate participants into the injured group. Other definitions of RRI have been used when medical evaluation was not part of the study design. These methods are based primarily on the number of missed or modified running days in a given week due to pain (Yamato et al., 2015), which was not available in the present study, while others use similar details to assess RRI severity (Salzler et al., 2016). A new consensus statement on sports injury surveillance suggests that no one definition of injury may fit all circumstances (Bahr et al., 2020). Given that complexity is sensitive to detecting differences between groups of varying health status and between disease severity levels, future development of COM complexity as an RRI surveillance system could lead to a data-driven definition for RRI that avoids self-report and subjective behavioral responses to pain as part of the criteria. RRI severity or type may also influence the degree of change in complexity magnitude between runs and should be investigated in future studies.

Six out of the seven injured participants in the present study reported the onset of their RRI within 3 weeks of the baseline run, including two participants who sustained a run 2–5 days after the baseline run. As such, only one run was included in the analysis for these two participants. The lack of time between the baseline run and the onset of an RRI may suggest that monitoring of the would-be injured participants did not begin early enough to capture a greater change in complexity. Recording more runs and longer follow-up periods may improve the classification accuracy of changes in complexity with the odds of sustaining an RRI.

Accelerometer placement and compliance were self-controlled by the participants, and study personnel were not present prior to any running bouts or practice other than the baseline run to ensure these procedures were followed consistently. However, accelerometer wear compliance exceeded the reported compliance of studies implementing a prescribed training program including a follow-up period of 4–24 weeks but was lower than shoe-related intervention studies with a 21–24-week follow-up (Nielsen et al., 2019) and another collegiate runner surveillance study with nine participants (Kiernan et al., 2018). Participants were also instructed to position the accelerometer securely, centered over the low back (which was demonstrated by the investigators prior to the baseline run). Self-placed accelerometers are a necessary part of any ecologically valid study. Calculating the Euclidean norm of the accelerometer data obviates the need to account for interindividual differences in device orientation. Any effects on signal quality caused by the degree of fixation was likely identified as “non-running” by the run detection algorithm (Davis IV et al., 2019), and thus removed from the analysis.

The low (i.e., 100 Hz) sampling frequency of the research-grade accelerometer used in this study is another limitation. While some wireless, research-grade wearable devices have greater sampling frequency capabilities, the accelerometer used in this study was chosen for its access to the raw data and for its data storage capacity (accelerometers were not returned to the researchers until the end of the competitive season, ~14 weeks after the baseline run). However, an underestimation analysis conducted by our laboratory revealed that peak COM acceleration recorded by the accelerometer used in this study and a higher resolution (1,200 Hz) differed by <1 g. This analysis suggests that the 100Hz sampling frequency was sufficient to assess COM acceleration complexity.

### Future Directions

Investigating the use of wearables for out-of-lab assessments of running mechanics has recently expanded to improve ecological validity in RRI research. Despite the added value of quantifying running mechanics and external loads experienced by runners while training (Napier et al., 2020; Paquette et al., 2020; Ryan et al., 2021), coaches and athletes have, for the most part, exclusively relied on running distance to minimize RRI risk due to convenience and simplicity. However, it is not typically revealed to a runner that a training error has occurred until after the signs and symptoms of an RRI manifest. The present study is the demonstrated first step in providing a monitoring or warning system to intervene before RRI onset. As the wearable technology industry solves current barriers and limitations for more advanced applications—including access to raw data, data storage capacity, and computing power—advancements in mathematical and statistical models to prospectively determine onset of RRI, such as those proposed in the current study, may become more feasible. The current study is in line with these future applications of wearable technology for coaching, rehabilitation, and prevention as it serves as a template for monitoring runners in a free-living setting.

### Conclusion

This pilot study establishes proof of concept and initial feasibility for using COM acceleration complexity as a metric for monitoring risk of RRI. The nearly consistent but non-significant estimates that increase in COM acceleration complexity which corresponded to increased odds of RRI risk in our pilot sample is worthy of investigation in larger studies. However, the true association between complexity and injury may be positive, negative, or null. Additional basic science and clinical investigations are required to reveal specific changes in gait complexity that may be indicative of RRI risk. This work could lead to coaching, rehabilitation, and recreational fitness strategies using wearable technology to monitor training and prevent RRI before its onset.

## Data Availability Statement

The raw data supporting the conclusions of this article will be made available by the authors upon request, without undue reservation.

## Ethics Statement

This study involving human participants was reviewed and approved by University of Memphis and Indiana University Institutional Review Boards. The participants provided their written informed consent to participate in this study.

## Author Contributions

AHG conceptualized the study and developed the research questions. Funding was obtained by MRP (principal investigator) and AHG (coinvestigator). AHG and MRP were responsible for the study design and oversight. Data collection and coordination with the athletic team were performed by MRP. Data analysis was conducted by JEV and JM. Custom MATLAB programs were written by JM, JJD, and JEV. The statistical analysis was designed by JH and JJD and was executed by JJD. AHG, JEV, JM, and JJD performed the initial drafting of the manuscript. All authors contributed to editing of the final manuscript and agree to be accountable for the content of the work.

## Conflict of Interest

The authors declare that the research was conducted in the absence of any commercial or financial relationships that could be construed as a potential conflict of interest.
