# Supplementary material for: Monitoring Gait Complexity as an Indicator for Running-Related Injury Risk in Collegiate Cross-Country Runners: A Proof-of-Concept Study
Source: Front Sports Act Living. 2021 May 21;3:630975. doi: 10.3389/fspor.2021.630975 (PMC8177207; doi:10.3389/fspor.2021.630975)
Supplement: Supplementary file 1 [file Data_Sheet_1.pdf]

Supplementary Material

1 Supplementary Figures

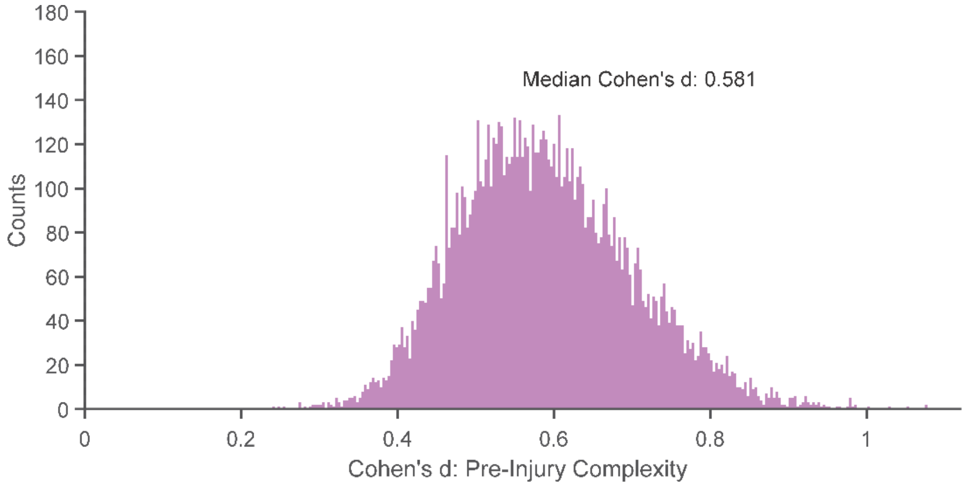

Supplementary Figure 1. Histogram showing distribution of Cohen's d effect sizes for group differences in pre-injury complexity across 10,000 replicates of the frequency-matching procedure. "Pre-injury" complexity was the last observed complexity value prior to reported injury in the injured group and the date-matched run of the control group resulting from the frequency-matching procedure. Positive effect sizes indicate greater complexity values in the injured group as compared to the uninjured group.

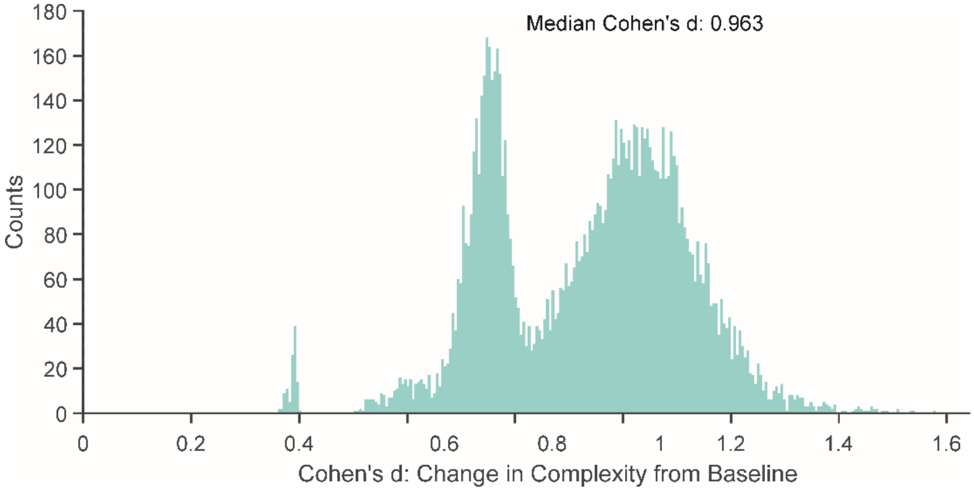

Supplementary Figure 2. Histogram showing distribution of Cohen's d effect sizes for group differences in change from baseline complexity (calculated as pre-injury complexity minus baseline complexity) across 10,000 replicates of the frequency-matching procedure. "Pre-injury" complexity was the last observed complexity value prior to reported injury in the injured group and the date-matched run of the control group resulting from the frequency-matching procedure.
